# Pembrolizumab-Induced Fatal Myasthenia, Myocarditis, and Myositis in a Patient with Metastatic Melanoma: Autopsy, Histological, and Immunohistochemical Findings—A Case Report and Literature Review

**DOI:** 10.3390/ijms241310919

**Published:** 2023-06-30

**Authors:** Elena Giovannini, Maria Paola Bonasoni, Michele D’Aleo, Ione Tamagnini, Matteo Tudini, Paolo Fais, Susi Pelotti

**Affiliations:** 1 Unit of Legal Medicine, Department of Medical and Surgical Sciences, University of Bologna, Via Irnerio 49, 40126 Bologna, Italy; elena.giovannini91@gmail.com (E.G.); michele.daleo@studio.unibo.it (M.D.); paolo.fais@unibo.it (P.F.); susi.pelotti@unibo.it (S.P.); 2 Pathology Unit, Azienda USL-IRCCS di Reggio Emilia, Via Amendola 2, 42122 Reggio Emilia, Italy; ione.tamagnini@ausl.re.it

**Keywords:** pembrolizumab, myasthenia, myocarditis, myositis, autopsy, immunohistochemistry

## Abstract

Immune checkpoint inhibitors (ICIs) represent a major advance in cancer treatment. The lowered immune tolerance induced by ICIs brought to light a series of immune-related adverse events (irAEs). Pembrolizumab belongs to the ICI class and is a humanized IgG4 anti-PD-1 antibody that blocks the interaction between PD-1 and PD-L1. The ICI-related irAEs involving various organ systems and myocarditis are uncommon (incidence of 0.04% to 1.14%), but they are associated with a high reported mortality. Unlike idiopathic inflammatory myositis, ICI-related myositis has been reported to frequently co-occur with myocarditis. The triad of myasthenia, myositis, and myocarditis must not be underestimated as they can rapidly deteriorate, leading to death. Herein we report a case of a patient with metastatic melanoma who fatally developed myasthenia gravis, myocarditis, and myositis, after a single cycle of pembrolizumab. Considering evidence from the literature review, autopsy, histological, and immunohistochemical investigations on heart and skeletal muscle are presented and discussed, also from a medical–legal perspective.

## 1. Introduction

Immune checkpoint inhibitors (ICIs) represent a major advance in cancer treatment, and they have significantly improved the survival of patients in many advanced malignancies, such as melanoma, non-small cell lung cancer, hepatocellular carcinoma, melanoma, renal cell carcinoma, and Hodgkin’s lymphoma [1,2,3].

In normal conditions, the immune response involves antigen-presenting cells (APCs), which process antigens and present them as peptide-major histocompatibility complexes to naive lymphocytes in secondary lymphoid organs. As a consequence, activated T lymphocytes clonally proliferate and differentiate toward TCD4+ helper or TCD8+ cytotoxic phenotypes. These effector cells will further migrate and recognize the antigens in their original location via T cell receptors (TCRs). In physiologic conditions, several mechanisms dampen the activation of T cells in order to prevent autoimmunity. For example, programmed cell death protein 1 (PD-1) expressed by activated T cells interacts with its ligand, programmed death-ligand 1 (PD-L1), which is expressed by APCs, lymphocytes, and epithelial cells in order to prevent aberrant immune reactions. In cancer, PD-L1 expression by tumors cells is a well-known mechanism of immune evasion. ICIs block these pathways and therefore restore T cell activity, decreasing immune tolerance and promoting autoimmunity [4,5,6]. Pembrolizumab belongs to the ICIs class and is a humanized IgG4 anti-PD-1 antibody that blocks the interaction between PD-1 and PD-L1 [7,8]. It is indicated in the treatment of various cancer subtypes, including metastatic melanoma [9]. It is associated with increased progression-free survival and overall survival in advanced melanoma [7,10,11,12].

The lowered immune tolerance induced by ICIs brought to light a series of immune-related adverse events (irAEs). Specifically, myocarditis is an uncommon irAE (incidence of 0.04% to 1.14%) but is associated with a high reported mortality (25% to 50%) [13,14,15]. The ICI-related irAEs involving various organ systems and myocarditis are the most common [3,4,5,9,13,14]. Although it may be silent with merely cardiac biomarker elevation, it may present as a fulminant disease, leading to cardiogenic shock and death [16]. ICI-related myositis is rare, accounting for less than 1% of irAEs, but has a high fatality rate of up to 22% [17,18]. Interestingly, unlike idiopathic inflammatory myositis, ICI-related myositis has been reported to frequently co-occur with myocarditis in up to 40% of cases [19,20].

Among the irAEs of pembrolizumab, myocarditis and neuromuscular adverse events are considered rare [8,13]. The triad of myasthenia, myositis, and myocarditis must not be underestimated as they can rapidly deteriorate, leading to death. As long as the symptoms present, they should be treated promptly with high doses of corticosteroids altogether with cardiac therapeutic support [21].

An adverse drug reaction is defined as a harmful or unpleasant reaction, resulting from a medicinal management. This event is imputable to medical liability when proper precautions and/or treatment were not carefully employed; therefore, it is related to a medical error if it was predictable and preventable. In contrast, when an adverse event was not preventable, the patient’s injuries do not result from medical error [22,23,24,25]. 

Herein we report a case of a patient with metastatic melanoma who fatally developed myasthenia gravis, myocarditis, and myositis, after a single cycle of pembrolizumab. The patient’s relatives requested post-mortem investigations in suspicion of professional medical liability. Autopsy, histological, and immunohistochemical investigations on the heart and skeletal muscle are presented and discussed, also from a medical–legal perspective. 

Similar cases reported in the literature, presenting myasthenia gravis, myositis, and myocarditis, have also been reviewed and compared. 

## 2. Case Presentation

### 2.1. Clinical Evolution

A 65-year-old male presented a nodular melanoma of the left forearm. After the excision, the histopathological examination reported the following parameters: no ulceration, no regression, Breslow thickness of 12 mm, mitoses for 10 mm^2^, vertical growth phase, moderate presence of tumor infiltrating lymphocytes (TIL) of “non-brisk type”, microsatellites and lymphovascular involvement not identifiable, and neoplastic infiltration of the deep surgical margin. The histopathologic stage was pT4a > 4.0 mm [26]. 

The molecular profile detected many *NRAS* mutations: Q61K (c.181 C>A), Q61R (c.182 A>G), Q61L (c.182 A>T), and Q61H (c.183 A>T). 

An ultrasound (US) of the left axilla revealed a hypoechoic, highly vascularized lymph node of 10 mm, metabolically active at positron emission tomography (PET). A US-guided fine needle aspiration biopsy confirmed the clinical diagnosis of metastatic melanoma. After one month of the initial diagnosis, the patient underwent widening of the excision margins and left axillary lymphadenectomy. The histological analysis of the skin resulted negative for neoplasia, but metastatic melanoma with extracapsular extension was found in one in four of the examined lymph nodes (pN1b) [26]. 

As the patient was in good clinical conditions, he started adjuvant immunotherapy with pembrolizumab (200 mg every three weeks), after approximately 3 months of the histological diagnosis of skin melanoma. 

After 12 days from the first dose, the patient presented fever (37.8 °C), constipation, and muscle aches. Symptoms progressively worsened with diplopia and gait instability, and after 15 days from the first dose, he was admitted to the hospital. The neurological examination detected impaired lateral rotation of the right eye and decreased strength in the lower limbs. Myasthenia and myositis were suspected, and then he was then started with intravenous methylprednisolone 60 mg, one dose in the morning, and oral pyridostigmine 60 mg three times a day. An electrocardiogram (ECG) showed sinus rhythm with a right bundle branch block. Laboratory blood tests evidenced acute kidney failure with serum creatinine of 2.49 mg/dL and glomerular filtration rate (GFR) of 23 mL/min/1.73 m^2^. Alanine and aspartate Transaminases (ALT and AST) were elevated with values of 659 and 1687 U/L, respectively. C reactive protein (CRP) was also increased with 7.9 mg/dL. 

On the second day of hospitalization, the patient complained of dyspnea and palpitation. ECG showed atrial fibrillation, and the echocardiogram showed a non-dilated left ventricle with ejection fraction > 60%. Anticoagulant therapy was started. Neurologic symptoms persisted with the development of left eyelid ptosis. Myocarditis was suspected as high levels of troponin I (25,010 ng/L) and creatine kinase (14,977 U/L) were found. The day after, cardiological symptoms worsened with atrioventricular block second-degree Mobitz type 2. Due to respiratory and renal failure, non-invasive ventilation with FiO_2_ 40% and dialysis were started. However, severe hypoxemia and ventricular tachycardia with peak torsions fibrillation arose. 

The patient died despite resuscitative maneuvers, after 15 days from the first dose of pembrolizumab.

### 2.2. Autopsy Findings 

The external examination identified residual medical procedures, such as previous sites of injections, but it was otherwise unremarkable. 

Internal examinations detected bilateral serosanguinous pleural effusions (180 mL within the left pleural cavity and 135 mL in the right). The lung weight and volume were increased: the right lung was 1020 g, and the left was 800 g. On sectioning, the parenchyma was congested and compact, with a slight amount of red-brownish clear foamy fluid. The heart presented a globular shape with an increased weight of 530 g, and the diameters were longitudinal of 17 cm and transverse of 14 cm. At slicing, the left ventricle appeared marbled and dyschromic. Coronary artery disease with wall calcification diffusely involved all three vessels. The left main trunk showed severe atherosclerosis with 80% luminal occlusion. 

The liver weighed 1715 g with a yellowish cut surface. The kidneys (right kidney 240 g, left kidney 255 g) presented a brownish-red parenchyma with a faded cortico-medullary junction. 

The spleen (240 g) was heavily congested with scarce evidence of the white pulp.

No other significant findings were observed in the other organs. 

### 2.3. Histological Findings

**Figure 1:**
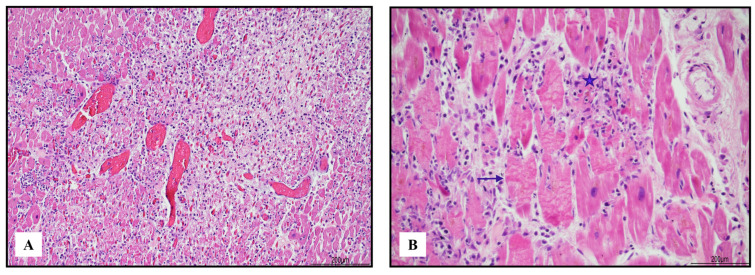
Severe lympho-histiocytic myocarditis (hematoxylin and eosin). Areas of damaged myocardium were diffuse and involved both ventricles and the septum ((**A**) 4 HPF). In those zones, necrosis (blue star) and contraction bands (blue arrow) were well represented ((**B**) 40 HPF).

In the heart, lympho-histiocytic myocarditis associated with myocardial necrosis and contraction bands was diffuse and severe, involving both ventricles, the septum, and the trabecular myocardium (Figure 1). 

Immunohistochemistry was performed on the heart and skeletal muscle. The antibodies utilized and their characteristics are summarized in Table 1.

**Figure 2:**
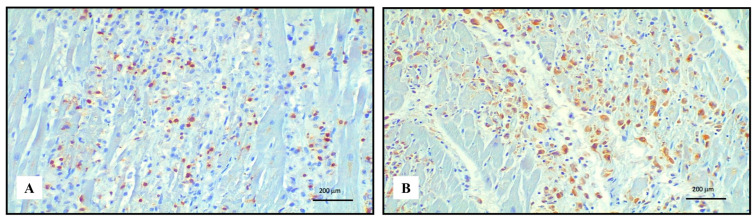
Immunohistochemistry of lympho-histiocytic myocarditis. Abundance of TCD3+ lymphocytes ((**A**) 20 HPF) and CD68 PGM1+ macrophages ((**B**) 20 HPF).

**Figure 3:**
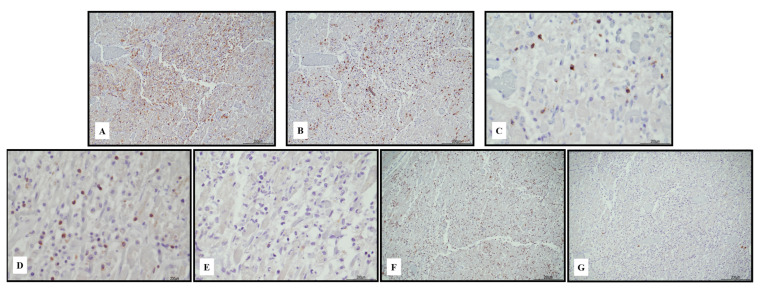
Immunohistochemical characterization of the lymphocytic inflammatory response in myocarditis. TCD4+ cells ((**A**) 10 HPF) were rare compared with TCD8+ ((**B**) 10 HPF), but there was a cross reaction with macrophages, and the darker brown cells were lymphocytes. TCD8+ cytotoxic cells were Tia-1+ ((**C**) 40 HPF) and PD-1+ ((**D**) 40 HPF), but Granzyme B negative ((**E**) 40 HPF). TCD25+ regulatory T cells were abundant ((**F**) 10 HPF), intermixed with rare BCD20+ lymphocytes ((**G**) 10 HPF). Scale bar: 200 μm.

**Figure 4:**
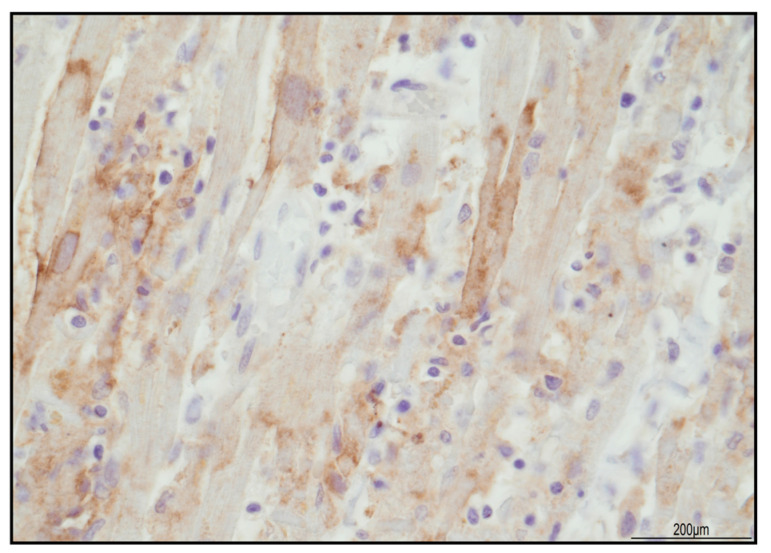
PD-L1 expression in the damaged areas was focally expressed in the cytoplasmic membrane of myocardiocytes (40 HPF).

**Figure 5:**
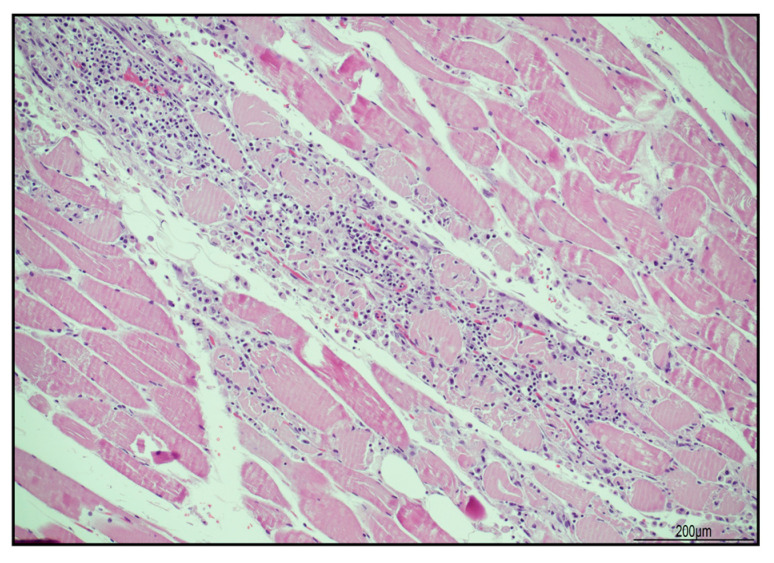
Necrotizing myositis (hematoxylin and eosin). Sample from iliopsoas muscle with abundant inflammatory infiltrate surrounding necrotic myofibers (20 HPF).

Immunohistochemistry of the inflammatory response revealed an abundance of TCD3+ lymphocytes and CD68 PGM1+ macrophages (Figure 2). Immunohistochemical features of lymphocytes revealed numerous TCD8+ cells outnumbering TCD4+. TCD8+ cytotoxic cells were Tia-1+ and PD-1+ but Granzyme B negative (Figure 3). TCD25+ regulatory T cells were well represented, but BCD20+ lymphocytes were scarce (Figure 4). PD-L1 was focally expressed within the myocardiocytes in the damaged areas (Figure 5). 

**Figure 6:**
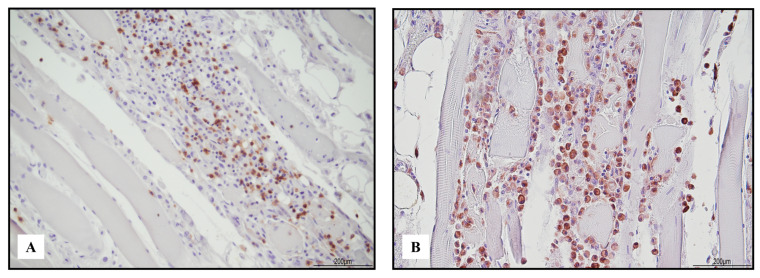
Immunohistochemical features of necrotizing myositis. Abundance of TCD3+ lymphocytes ((**A**) 10 HPF) and CD68 PGM1+ macrophages ((**B**) 20 HPF).

**Figure 7:**
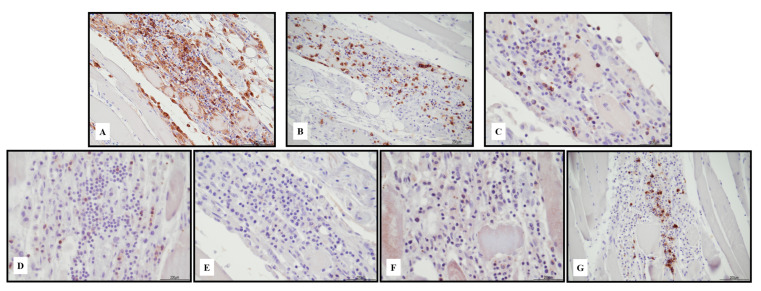
Immunohistochemical characterization of the lymphocytic inflammatory response in necrotizing myositis. TCD4+ cells ((**A**) 20 HPF) presented a cross reaction with macrophages, and lymphocytes were the darker brown. TCD4+ cells and TCD8+ ((**B**) 20 HPF) were in a similar ratio. TCD8+ cytotoxic cells were Tia-1+ ((**C**) 40 HPF) and PD-1+ ((**D**) 40 HPF), but Granzyme B negative ((**E**) 40 HPF). TCD25+ regulatory T cells were almost absent ((**F**) 40 HPF), associated with abundant BCD20+ lymphocytes ((**G**) 20 HPF). Scale bar: 200 μm.

**Figure 8:**
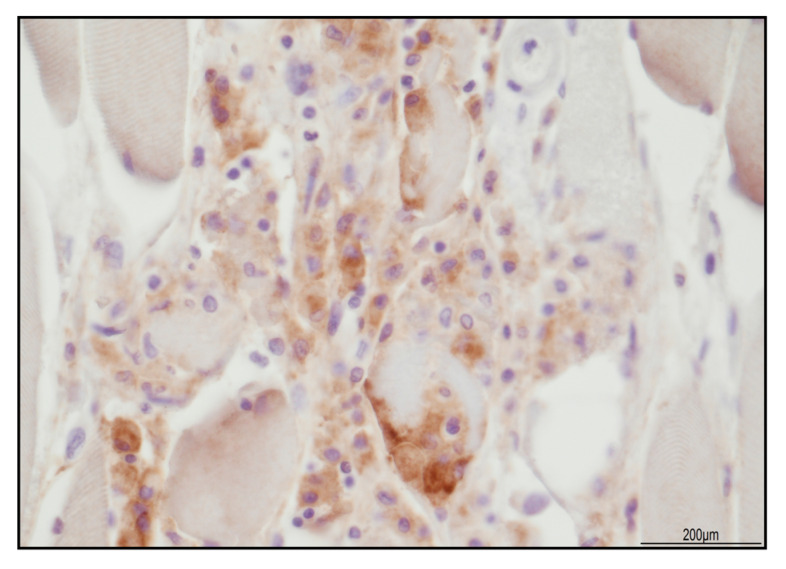
PD-L1 expression in necrotizing myositis. Focal positivity in the injured myocytes and within the macrophages (40 HPF).

The sample from the iliopsoas muscle displayed necrotizing myositis characterized by TCD3+ lymphocytes and CD68 PGM1+ macrophages (Figure 6). The TCD4+ and TCD8+ cells were in a similar ratio; TCD8 expressed Tia-1 and PD1, but Granzyme B was negative. TCD25+ lymphocytes were negative, but BCD20+ lymphocytes were abundant (Figure 7). PD-L1 was focally recognizable in the injured myocytes and within the macrophages (Figure 8). 

Regarding the other organs, in the right lung, there was a focus of broncopneumonia with neutrophils. In both lungs, leukocyte margination was observed. The liver showed centrolobular necrosis. In both kidneys, there was acute tubular necrosis and mild and focal pyelonephritis. 

### 2.4. Literature Review

An electronic search was performed in three databases (PubMed, Scopus, and Web of Science), and keywords related to the study aim and included in the search string were as follows: *pembrolizumab AND (myasthenia OR myositis) AND myocarditis AND melanoma*. The English language and time interval of publication, from January 1990 to March 2023, were applied as filters and inclusion criteria. 

The literature review showed that 16 cases of myocarditis and myositis following pembrolizumab administration have previously been published [5,18,27,28,29,30,31,32,33,34,35,36,37,38,39,40]. However, autopsies and histological analyses of heart and muscle were performed in only two patients [27,28]. In other four reports, cardiac and/or muscle biopsies were performed before death [29,30]. Touat et al. [31] revised a case series in which only one patient with melanoma developed symptoms after a single dose of ICU, but the data were aggregated, and no further information was possible to retrieve. All the data are summarized in Table 2. 

Of note, regarding the cases retrieved, the prevalence was of male subjects (11/15), and the age ranged from 25 to 83 years (the case described by Touat et al. [31] has not been included). In five patients, the treatment was fatal after a single dose, and an autopsy was performed only in two, confirming myocarditis and myositis [27,28]. In the case described by Martinez-Calle et al. [27], dyspnea and malaise developed after 16 days, and death occurred after 26 days from the dose. In the other case [28], palpitation and myalgia presented after 16 days and the patient died after 28 days. In the patient described by Portoles Hernández et al. [29], shortness of breath occurred after 10 days and death after 20. In the case reported by Soman et al. [32], left-sided partial ptosis and facial droop developed after 3 weeks, and death occurred in 28 days. Konstantina et al. [33] reported the shortest time, 3 days, between drug administration and symptom onset with chest pain and muscle weakness; death occurred after 67 days. The other three fatal cases presented myositis and myocarditis-related symptoms after the second dose of the drug [30,34,35]. Histology was available in the autopsy cases [27,28], and in one patient, biopsies on heart and muscle were performed before death [30]. In the other deceased case [29], myocarditis was histologically confirmed, but myositis was only diagnosed clinically. In two other patients, who survived the irAEs, biopsies confirmed myocarditis and myositis [18] and myositis only [36]. 

## 3. Discussion

We described a fatal case of a 65-year-old male who developed myasthenia, myocarditis, and myositis after a single dose of pembrolizumab.

Our patient presented with fever and muscle pain 12 days after the first dose of pembrolizumab. The symptoms progressively worsened with diplopia, gait instability, impaired eye movements, ptosis, and lower limb myasthenia. Contemporarily, myocarditis developed with atrioventricular block second-degree Mobitz type 2 and high levels of troponin I (25,010 ng/L) and creatine kinase (14,977 U/L). Death occurred after 15 days from the first ICI dose. On the whole, these signs and symptoms were in alignment with those previously reported in the literature, especially the development of muscle compromise first and then the occurrence of myocarditis. In this condition, biomarkers such as cardiac troponin T, creatine kinase, and transaminase levels are typically elevated [4,13,20,27,30,35,39,41]. Moreover, a complete atrioventricular block and a preserved left ventricular systolic function is usually shown by ECG and echocardiogram [20,39,41]. Similarly, pembrolizumab-related systemic myositis involving the ocular muscles resembles myasthenia gravis, but its related antibodies are absent [34,35,37,42]. 

Histologically, in our case we found evidence of lympho-histiocytic myocarditis and necrotizing myositis, but other organs were not affected. 

In myocarditis, immunohistochemistry evidenced an abundance of CD68 PGM1+ macrophages associated with TCD8+/Tia-1+/PD-1+. TCD25+ regulatory cells were present, but BCD20+ lymphocytes were rare. In the damaged areas, PD-L1 was focally expressed within the myocardiocytes but not in the uninvolved myocardium. The typical ICI-related myocarditis is characterized by TCD8+ cell infiltration and upregulation of PDL-1 in the areas of necrosis, as observed in our patient [14,43,44]. PD-1 may result as positive in TCD8 cells or negative, indicating receptor engagement by the drug [27,44]. TCD8+ cells surrounding PD-L1+ myocardiocytes may favor a targeted autoimmune response likely due to PD-1 blockade [45]. Martinez-Calle et al. [27] described myocardial inflammation of TCD8+/Tia-1+/PD-1− cells, but found no multiorgan damage, to the contrary of what was described by Fuentes-Antrás et al. [28]. BCD20 negativity associated with granulation tissue after immunosuppression was also reported [46]. 

Regarding necrotizing myositis, in our patient, it was composed of TCD3+ cells, with a similar ratio of TCD4+ and TCD8+/Tia-1+/PD-1+. PD-L1 was positive within the damaged myocytes and in the surrounding CD68 PGM1+ macrophages. Compared with the myocardium, BCD20+ cells were abundant, but TCD25+ cells were negative. Necrotizing myositis after ICI treatment has been reported, composed of TCD8+/PD-1+ and CD68+ macrophages [31,43]. Although our findings were in agreement with the literature, in our patient, TCD4+ and TCD8+ were in a similar ratio. This finding may be explained as a limited anti-PD-1 impairment therapy, as also described in a patient treated with nivolumab [47]. Anti-PD-1 antibodies favor an increase in TCD8+ because the PD-1/PD-L1 axis prevents T cell activation; therefore, their block facilitates T cytotoxic enrollment. On the other hand, TCD4+ excess recruitment has been described more in association with anti-CTLA-4 therapy [48]. In our case, PD-L1 was seen in macrophages, consistently with previous findings on iRAE myositis [49]. Moreover, the presence of PD-L1 on myofiber and PD-1+ lymphocytes may suggest a potential interaction of the PD-1/PD-L1 pathway in regulating T cell activity [49].

In general, in the reported cases treated with pembrolizumab and further development of myocarditis and myositis, histological assessments, including immunohistochemistry, are scarcely examined in depth, with the exception of Martinez-Calle et al. [27]. In two other cases, myocarditis and myositis were mentioned with lymphocytic infiltration only [30,36]. Thus, the findings in our case were compared with other ICI drugs [31,43,47,48,49]. For pembrolizumab, we summarized the available information in Table 3. 

In order to assess the causal link between the administration of the pembrolizumab and the patient’s death, medico-legal criteria were evaluated. First, myocarditis and myositis are recognized as adverse events of pembrolizumab and in our case were diagnosed clinically and confirmed at autopsy. The onset of symptoms was 12 days after the administration of pembrolizumab, and this occurrence agrees with the literature data, which include a period ranging between one and four weeks. Moreover, our histological observations were in agreement with previous descriptions, although scarcely reported. In our case, the management of the patient, including the modality and timing of drug administration and the prompt treatment of adverse effects, was coherent with the guidelines [50,51]. Therefore, myocarditis and myositis were considered adverse effects of the drug, predictable but not preventable. The area of understanding how irAEs work is not extensively researched, and autoimmunity is becoming a significant challenge for ICI therapies [52,53]. Approximately 43.6% of cancer patients in the United States are eligible for ICI therapies. Unfortunately, most of these patients will experience irAEs without benefiting from ICI therapies [54]. Since both anti-CTLA-4 and anti-PD-1 antibodies are not specific to tumors, it has been suggested that the development of irAEs is linked to disturbances in self-tolerance. In fact, irAEs share similarities with chronic graft-versus-host disease (GVHD) reactions observed after allogeneic bone marrow transplantation. This concept has led to the exploration of lower doses of ICI drugs. By utilizing an off-label approach with a low-dose combination of anti-CTLA-4 and anti-PD-1 antibodies, it has been demonstrated that harnessing autoimmune mechanisms is safer than established protocols while maintaining effectiveness in cancer patients who have exhausted conventional treatments [52]. In fact, a study involving 131 stage IV cancer patients with 23 different cancer types, all of whom had exhausted conventional treatments, showed that our off-label low-dose protocol of ipilimumab (0.3 mg/kg) and nivolumab (0.5 mg/kg) along with other T cell stimulation methods was significantly safer than approved protocols without compromising efficacy [52].

## 4. Conclusions

ICI immunotherapy represents an encouraging treatment for advanced tumors; however, adverse effects affecting multiple organs and systems are still a major issue with this therapy. Despite their rarity, myocarditis and myositis are documented adverse and potentially fatal effects of pembrolizumab, but the histological and immunohistochemical findings of these lesions are still scarcely reported in the literature. The extension of casuistry and implementation of further investigations are required, especially on the molecular level, to better understand the triggered autoimmunity after the different classes of ICIs and the appropriate treatment.

## Figures and Tables

**Table 1:** Antibodies utilized in immunohistochemistry on heart and skeletal muscle.

Antibody	Clone	Company
CD3	2GV6 Rabbit Monoclonal	Ventana Group, Milan, Italy
CD4	SP35 Rabbit Monoclonal	Ventana Group, Milan, Italy
CD8	SP57 Rabbit Monoclonal	Ventana Group, Milan, Italy
CD20	L26 Mouse Monoclonal	Ventana Group, Milan, Italy
CD25	4C9 Mouse Monoclonal	Cell Marque, Rocklin, CA, USA
CD68	PGM1 Mouse Monoclonal	DBS, Pleasanton, CA, USA
Granzyme B	Rabbit Polyclonal	Cell Marque, Rocklin, CA, USA
Tia-1	2G9A10F5 Mouse Monoclonal	Bio-Genex, Fremont, CA, USA
PD-1	NAT105 Mouse Monoclonal	Ventana Group, Milan, Italy
PD-L1	22c3 Mouse Monoclonal	Dako Agilent, Santa Clara, CA, USA

**Table 2:** Cases of myocarditis and myositis following pembrolizumab administration.

Articles	Pazient Age and Sex (M, Male; F, Female)	Advanced Neoplasia Treated	Pembrolizumab Number of Doses Administered	Timing and Onset Symptoms after Last Dose	Dead (D) or Alive (A): Number of Days Hospitalized (H) after the Dose Administration	Autopsy Performed (Yes or No) and Histological Findings	Biopsy (Yes or No) and Histological Findings
Nguyen et al. [18]	25, M	Thymoma	1	14 days: chest pain, subtle myalgia	A: 47 days (33 days H)	No	Yes: myositis and myocarditis
Martinez-Calle et al. [27]	67, F	Myeloma	1	16 days: dyspnea malaise	D: 26 days (10 days H)	Yes: myositis and myocarditis	No
Fuentes-Antrás et al. [28]	75, M	Lung Adenocarcinoma	1	21 days: palpitation, myalgia	D: 28 days (7 days H)	Yes: myositis and myocarditis	No
Portoles Hernández et al. [29]	48, F	Thymoma	1	10 days, shortness of breath and muscle weakness	D: 20 days (10 in H)	No	Yes: myocarditis
Matsui et al. [30]	69, M	Urothelial carcinoma	2	5 days: severe fatigue abnormal gait	D: 22 days (17 days H)	No	Yes: myocarditis (HLA-positive cells on myofiber in muscle bundles)
Touat et al. [31]	65, M	Melanoma	1	12 days: fever, muscle aches, constipation, diplopia, gait instability	D: 15 days	Yes: myositis and myocarditis	No
Soman et al. [32]	70, F	Lung adenocarcinoma	1	21 days: left-sided partial ptosis and facial droop	D: 28 days (7 days H)	No	No
Konstantina et al. [33]	30, F	thymoma	1	3 days: chest pain and muscle weakness	D: 67 (64 H)	No	No
Hellman et al. [34]	83, M	Urothelial Carcinoma	2	4 days: ophtalmoplegia	D: 13 days after the second dose (9 days H)	No	No
Nasr et al. [35]	79, M	Gastric adenocarcinoma	2	14 days: ptosis, ophtalmoplegia	D: 22 days (8 days H)	No	No
Nagakomi et al. [5]	77, M	Renal cell Carcinoma	1	21 days: hoarseness and back pain	A: after 80 days (59 days H)	No	No
Xie et al. [36]	67, M	Lung neuroendocrine carcinoma	1	14 days: dyspnea on exertion, ptosis, blurred vision, and quadriparesis	A: myocarditis myositis and after 57 days H, immune-related pneumonia	No	Yes: myositis with lymphocyte infiltration
Sanchez-Sancho et al. [37]	63, M	Liposarcoma	1	6 days: diplopia, palpebral ptosis, myalgia predominating in the lower extremities, dysphagia, dysphonia, left ventricular ejection fraction 40%	A: 41 days (35 days after H myasteniform dysphagia and clinical symptoms)	No	No
Cao et al. [38]	69, M	Esophagogastrci carcinoma	1	14 days: Stevens-Johnson syndrome/toxic epidermal necrolysis (SJS/TEN) 2 months: limb weakness and shortness of breath	A: 74 days (recovered after 60 days H)	No	No
Todo et al. [39]	63, M	Urothelial carcinoma	1	12 days: left ptosis, diplopia	A: 321 days H	No	No
Shirai et al. [40]	83, M	Melanoma	1	25 days: fatigable weakness and muscle pain	A: 75 days (50 days H)	No	No

**Table 3:** Summary of the immunohistochemical available data in the patients reported in the literature treated with pembrolizumab.

		Articles					
		Nguyen et al. [18]	Martinez-Calle et al. [27]	Fuentes-Antrás et al. [28]	Portoles Hernández et al. [29]	Imai et al. [46]	Current Case
Heart	*CD3*	+++	+++	+++	+++	+++	+++
	*CD4*		+				+
	*CD8*			+++		+++	+++
	*CD20*					-	-/+
	*CD25*						+++
	*CD68*	+++	+++	+++		+++	+++
	*Granzyme B*						-
	*Tia-1*		+ in CD8				+ in CD8
	*PD-1*						+
	*PD-L1*						+ only in damaged myocardiocytes
Skeletal muscle	*CD3*	+++	+++	+++			+++
	*CD4*						+
	*CD8*			+++			+
	*CD20*						+++
	*CD25*						-
	*CD68*	+++	+++	+++			+++
	*Granzyme B*						-
	*Tia-1*		+ in CD8				+ in CD8
	*PD-1*		-				+ in CD8
	*PD-L1*						+ in injured myocytes and macrophages

Legend: +++ abundant; + present; -/+ rare; - negative.

## Data Availability

The data presented in this study are available on request from the corresponding author.

## References

[1] Nardi Agmon I., Itzhaki Ben Zadok O., Kornowski R. (2022). The Potential Cardiotoxicity of Immune Checkpoint Inhibitors. J. Clin. Med..

[2] Ziogas D.C., Theocharopoulos C., Koutouratsas T., Haanen J., Gogas H. (2022). Mechanisms of resistance to immune checkpoint inhibitors in melanoma: What we have to overcome?. Cancer Treat. Rev..

[3] Chen Y., Jia Y., Liu Q., Shen Y., Zhu H., Dong X., Huang J., Lu J., Yin Q. (2021). Myocarditis related to immune checkpoint inhibitors treatment: Two case reports and literature review. Ann. Palliat. Med..

[4] Cozma A., Sporis N.D., Lazar A.L., Buruiana A., Ganea A.M., Malinescu T.V., Berechet B.M., Fodor A., Sitar-Taut A.V., Vlad V.C. (2022). Cardiac Toxicity Associated with Immune Checkpoint Inhibitors: A Systematic Review. Int. J. Mol. Sci..

[5] Nakagomi Y., Tajiri K., Shimada S., Li S., Inoue K., Murakata Y., Murata M., Sakai S., Sato K., Ieda M. (2022). Immune Checkpoint Inhibitor-Related Myositis Overlapping With Myocarditis: An Institutional Case Series and a Systematic Review of Literature. Front. Pharmacol..

[6] Rasmussen M., Durhuus J.A., Nilbert M., Andersen O., Therkildsen C. (2022). Response to Immune Checkpoint Inhibitors Is Affected by Deregulations in the Antigen Presentation Machinery: A Systematic Review and Meta-Analysis. J. Clin. Med..

[7] Kwok G., Yau T.C., Chiu J.W., Tse E., Kwong Y.L. (2016). Pembrolizumab (Keytruda). Hum. Vaccines Immunother..

[8] Schiopu S., Käsmann L., Schönermarck U., Fischereder M., Grabmaier U., Manapov F., Rauch J., Orban M. (2021). Pembrolizumab-induced myocarditis in a patient with malignant mesothelioma: Plasma exchange as a successful emerging therapy-case report. Transl. Lung Cancer Res..

[9] Darnell E.P., Mooradian M.J., Baruch E.N., Yilmaz M., Reynolds K.L. (2020). Immune-Related Adverse Events (irAEs): Diagnosis, Management, and Clinical Pearls. Curr. Oncol. Rep..

[10] Eggermont A., Blank C.U., Mandala M., Long G.V., Atkinson V., Dalle S., Haydon A., Lichinitser M., Khattak A., Carlino M.S. (2018). Adjuvant Pembrolizumab versus Placebo in Resected Stage III Melanoma. N. Engl. J. Med..

[11] Hamid O., Robert C., Daud A., Hodi F.S., Hwu W.J., Kefford R., Wolchok J.D., Hersey P., Joseph R., Weber J.S. (2019). Five-year survival outcomes for patients with advanced melanoma treated with pembrolizumab in KEYNOTE-001. Ann. Oncol..

[12] Robert C., Schachter J., Long G.V., Arance A., Grob J.J., Mortier L., Daud A., Carlino M.S., McNeil C., Lotem M. (2015). Pembrolizumab versus Ipilimumab in Advanced Melanoma. N. Engl. J. Med..

[13] Chen Q., Huang D.S., Zhang L.W., Li Y.Q., Wang H.W., Liu H. (2018). Fatal myocarditis and rhabdomyolysis induced by nivolumab during the treatment of type B3 thymoma. Clin. Toxicol..

[14] Palaskas N., Lopez-Mattei J., Durand J.B., Iliescu C., Deswal A. (2020). Immune Checkpoint Inhibitor Myocarditis: Pathophysiological Characteristics, Diagnosis, and Treatment. J. Am. Heart Assoc..

[15] Mahmood S.S., Fradley M.G., Cohen J.V., Nohria A., Reynolds K.L., Heinzerling L.M., Sullivan R.J., Damrongwatanasuk R., Chen C.L., Gupta D. (2018). Myocarditis in Patients Treated With Immune Checkpoint Inhibitors. J. Am. Coll. Cardiol..

[16] Ronen D., Bsoul A., Lotem M., Abedat S., Yarkoni M., Amir O., Asleh R. (2022). Exploring the Mechanisms Underlying the Cardiotoxic Effects of Immune Checkpoint Inhibitor Therapies. Vaccines.

[17] Allenbach Y., Anquetil C., Manouchehri A., Benveniste O., Lambotte O., Lebrun-Vignes B. (2020). Immune Checkpoint Inhibitor-Induced Myositis, the Earliest and Most Lethal Complication Among Rheumatic and Musculoskeletal Toxicities. Autoimmun. Rev..

[18] Nguyen L.S., Bretagne M., Arrondeau J., Zahr N., Ederhy S., Abbar B., Pinna B., Allenbach Y., Mira J.P., Moslehi J. (2022). Reversal of immune-checkpoint inhibitor fulminant myocarditis using personalized-dose-adjusted abatacept and ruxolitinib: Proof of concept. J. Immunother. Cancer.

[19] Aldrich J., Pundole X., Tummala S., Palaskas N., Andersen C.R., Shoukier M. (2021). Inflammatory Myositis in Cancer Patients Receiving Immune Checkpoint Inhibitors. Arthritis Rheumatol..

[20] Hamada N., Maeda A., Takase-Minegishi K., Kirino Y., Sugiyama Y., Namkoong H. (2021). Incidence and Distinct Features of Immune Checkpoint Inhibitor-Related Myositis from Idiopathic Inflammatory Myositis: A Single-Center Experience with Systematic Literature Review and Meta- Analysis. Front. Immunol..

[21] Ganatra S., Neilan T.G. (2018). Immune checkpoint inhibitor-associated myocarditis. Oncologist.

[22] Powell S.K. (2006). When things go wrong: Responding to adverse events: A consensus statement of the Harvard hospitals. Lippincotts Case Manag..

[23] Hofer T.P., Kerr E.A., Hayward R.A. (2000). What is an error?. Eff. Clin. Pract..

[24] Skelly C.L., Cassagnol M., Munakomi S. (2023). Adverse Events. StatPearls [Internet].

[25] Madea B. (2009). Medico-legal autopsies as a source of information to improve patient safety. Leg. Med..

[26] Keung E.Z., Gershenwald J.E. (2018). The eighth edition American Joint Committee on Cancer (AJCC) melanoma staging system: Implications for melanoma treatment and care. Expert Rev. Anticancer. Ther..

[27] Martinez-Calle N., Rodriguez-Otero P., Villar S., Mejías L., Melero I., Prosper F., Marinello P., Paiva B., Idoate M., San-Miguel J. (2018). Anti-PD1 associated fulminant myocarditis after a single pembrolizumab dose: The role of occult pre-existing autoimmunity. Haematologica.

[28] Fuentes-Antrás J., Peinado P., Guevara-Hoyer K., Díaz Del Arco C., Sánchez-Ramón S., Aguado C. (2022). Fatal Autoimmune Storm After a Single Cycle of Anti-PD-1 Therapy: A Case of Lethal Toxicity but Pathological Complete Response in Metastatic Lung Adenocarcinoma. Hematol. Oncol. Stem Cell Ther..

[29] Portolés Hernández A., Blanco Clemente M., Escribano García D., Velasco Calvo R., Núñez García B., Oteo Domínguez J.F., Salas Antón C., Méndez García M., Segovia Cubero J., Domínguez F. (2021). Checkpoint inhibitor-induced fulminant myo-carditis, complete atrioventricular block and myasthenia gravis—A case report. Cardiovasc. Diagn. Ther..

[30] Matsui H., Kawai T., Sato Y., Ishida J., Kadowaki H., Akiyama Y., Yamada Y., Nakamura M., Yamada D., Akazawa H. (2020). A Fatal Case of Myocarditis Following Myositis Induced by Pembrolizumab Treatment for Metastatic Upper Urinary Tract Urothelial Carcinoma. Internat. Heart J..

[31] Touat M., Maisonobe T., Knauss S., Ben Hadj Salem O., Hervier B., Auré K., Szwebel T.A., Kramkimel N., Lethrosne C., Bruch J.F. (2018). Immune checkpoint inhibitor-related myositis and myocarditis in patients with cancer. Neurology.

[32] Soman B., Dias M.C., Rizvi S.A.J., Kardos A. (2022). Myasthenia gravis, myositis and myocarditis: A fatal triad of immune-related adverse effect of immune checkpoint inhibitor treatment. BMJ Case Rep..

[33] Konstantina T., Konstantinos R., Anastasios K., Anastasia M., Eleni L., Ioannis S., Sofia A., Dimitris M. (2019). Fatal adverse events in two thymoma patients treated with anti-PD-1 immune check point inhibitor and literature review. Lung Cancer.

[34] Hellman J.B., Traynis I., Lin L.K. (2019). Pembrolizumab and epacadostat induced fatal myocarditis and myositis presenting as a case of ptosis and ophthalmoplegia. Orbit.

[35] Nasr F., El Rassy E., Maalouf G., Azar C., Haddad F., Helou J., Robert C. (2018). Severe ophthalmoplegia and myocarditis following the administration of pembrolizumab. Eur. J. Cancer.

[36] Xie X., Wang F., Qin Y., Lin X., Xie Z., Liu M., Ouyang M., Luo B., Gu Y., Li S. (2021). Case Report: Fatal Multiorgan Failure and Heterochronous Pneumonitis Following Pembrolizumab Treatment in a Patient with Large-Cell Neuroendocrine Carcinoma of Lung. Front. Pharmacol..

[37] Sanchez-Sancho P., Selva-O’Callaghan A., Trallero-Araguás E., Ros J., Montoro B. (2021). Myositis and myasteniform syndrome related to pembrolizumab. BMJ Case Rep..

[38] Cao J., Li Q., Zhi X., Yang F., Zhu W., Zhou T., Hou X., Chen D. (2021). Pembrolizumab-induced autoimmune Stevens-Johnson syndrome/toxic epidermal necrolysis with myositis and myocarditis in a patient with esophagogastric junction carcinoma: A case report. Transl. Cancer Res..

[39] Todo M., Kaneko G., Shirotake S., Shimada Y., Nakano S., Okabe T., Ishikawa S., Oyama M., Nishimoto K. (2019). Pembrolizumab-induced myasthenia gravis with myositis and presumable myocarditis in a patient with bladder cancer. IJU Case Rep..

[40] Shirai T., Kiniwa Y., Sato R., Sano T., Nakamura K., Mikoshiba Y., Ohashi N., Sekijima Y., Okuyama R. (2019). Presence of antibodies to striated muscle and acetylcholine receptor in association with occurrence of myasthenia gravis with myositis and myocarditis in a patient with melanoma treated with an anti-programmed death 1 antibody. Eur. J. Cancer.

[41] Katsume Y., Isawa T., Toi Y., Fukuda R., Kondo Y., Sugawara S., Ootomo T. (2018). Complete Atrioventricular Block Associated with Pembrolizumab-induced Acute Myocarditis: The Need for Close Cardiac Monitoring. Intern. Med..

[42] Kamo H., Hatano T., Kanai K., Aoki N., Kamiyama D., Yokoyama K., Takanashi M., Yamashita Y., Shimo Y., Hattori N. (2019). Pembrolizumab-related systemic myositis involving ocular and hindneck muscles resembling myasthenic gravis: A case report. BMC Neurol..

[43] Johnson D.B., Balko J.M., Compton M.L. (2016). Fulminant Myocarditis with Combination Immune Checkpoint Blockade. N. Engl. J. Med..

[44] Pradhan R., Nautiyal A., Singh S. (2019). Diagnosis of immune checkpoint inhibitor-associated myocarditis: A systematic review. Int. J. Cardiol..

[45] Sobol I., Chen C.L., Mahmood S.S., Borczuk A.C. (2020). Histopathologic Characterization of Myocarditis Associated With Immune Checkpoint Inhibitor Therapy. Arch. Pathol. Lab. Med..

[46] Imai R., Ono M., Nishimura N., Suzuki K., Komiyama N., Tamura T. (2019). Fulminant Myocarditis Caused by an Immune Checkpoint Inhibitor: A Case Report With Pathologic Findings. J. Thorac. Oncol..

[47] Vermeulen L., Depuydt C.E., Weckx P. (2020). Myositis as a neuromuscular complication of immune checkpoint inhibitors. Acta Neurol. Belg..

[48] Coutzac C., Adam J., Soularue E., Collins M., Racine A., Mussini C., Boselli L., Kamsukom N., Mateus C. (2017). Colon immune-related adverse events: Anti-CTLA-4 and anti-PD-1 blockade induce dis- tinct Immunopathological entities. J. Crohns Colitis..

[49] Knauss S., Preusse C., Allenbach Y., Leonard-Louis S., Touat M., Fischer N., Radbruch H., Mothes R., Matyash V., Böhmerle W. (2019). PD1 pathway in immune-mediated myopathies: Pathogenesis of dysfunctional T cells revisited. Neurol. Neuroimmunol. Neuroinflamm..

[50] Seth R., Messersmith H., Kaur V., Kirkwood J.M., Kudchadkar R., McQuade J.L., Provenzano A., Swami U., Weber J. (2020). Systemic Therapy for Melanoma: ASCO Guideline. J. Clin. Oncol..

[51] Garbe C., Amaral T., Peris K., Hauschild A., Arenberger P., Basset-Seguin N., Bastholt L., Bataille V., Del Marmol V., Dréno B. (2022). European consensus-based interdisciplinary guideline for melanoma. Part 2: Treatment—Update 2022. Eur. J. Cancer.

[52] Kleef R., Nagy R., Baierl A., Bacher V., Bojar H., McKee D.L., Moss R., Thoennissen N.H., Szász M., Bakacs T. (2021). Low-dose ipilimumab plus nivolumab combined with IL-2 and hyperthermia in cancer patients with advanced disease: Exploratory findings of a case series of 131 stage IV cancers—A retrospective study of a single institution. Cancer Immunol. Immunother..

[53] June C.H., Warshauer J.T., Bluestone J.A. (2017). Is autoimmunity the Achilles’ heel of cancer immunotherapy?. Nat. Med..

[54] Haslam A., Gill J., Prasad V. (2020). Estimation of the Percentage of US Patients With Cancer Who Are Eligible for Immune Checkpoint Inhibitor Drugs. JAMA Netw. Open.

